# Strong infrared photoluminescence in highly porous layers of large faceted Si crystalline nanoparticles

**DOI:** 10.1038/srep25664

**Published:** 2016-05-24

**Authors:** E. M. L. D de Jong, G. Mannino, A. Alberti, R. Ruggeri, M. Italia, F. Zontone, Y. Chushkin, A. R. Pennisi, T. Gregorkiewicz, G. Faraci

**Affiliations:** 1 Van der Waals – Zeeman Institute, University of Amsterdam, Science Park 904, 1098 XH Amsterdam, The Netherlands; 2 IMM – Consiglio Nazionale delle Ricerche (CNR-IMM), VIII Strada n°5 Zona Industriale, 95121 Catania, Italy; 3 ESRF, the European Synchrotron, CS40220, 38043 Grenoble Cedex 9, France; 4 Dipartimento di Fisica e Astronomia, Università di Catania, Via Santa Sofia 64, 95123 Catania, Italy

## Abstract

Almost all physical processes in solids are influenced by phonons, but their effect is frequently overlooked. In this paper, we investigate the photoluminescence of large silicon nanoparticles (approximately 100 nm size, synthesized by chemical vapor deposition) in the visible to the infrared detection range. We find that upon increasing laser irradiance, an enormous photoluminescence emission band appears in the infrared. Its intensity exhibits a superlinear power dependence, increasing over four orders of magnitude in the investigated pump power range. Particles of different sizes as well as different shapes in porous layers are investigated. The results are discussed taking into account the efficient generation of phonons under high-power pumping, and the reduced capability, porosity dependent, of the silicon nanoparticles to exchange energy with each other and with the substrate. Our findings are relevant for heat management strategies in silicon.

Crystalline silicon (Si) is a very attractive material and the backbone of electronic and photovoltaic industries. This is due to some key advantages, which include natural abundance, stability and nontoxicity, among others. More recently, a lot of research is dedicated towards sub 10 nm Si nanocrystals for which quantum confinement effects and surface prevalence play major roles. In particular, quantum confinement increases the band gap energy^1^ and induces also some other attractive electronic and optical properties. This research has opened important new perspectives for applications of Si nanocrystals^2^ for, e.g., visible optoelectronics^3^,^4^,^5^,^6^, photovoltaics^7^,^8^,^9^,^10^,^11^ and in medicine^12^. In spite of the impressive progress, size definition represents a major challenge and low-temperature production of Si nanocrystals with truly narrow size distribution remains elusive. In contrast, the development of nearly monodispersed ensembles of larger Si nanoparticles (NP’s), with dimensions in the tens to hundreds of nanometers range, has recently been reported^13^,^14^. These materials, requiring a temperature of only 50 °C for their production, offer an interesting combination of bulk Si electronic properties (no quantum confinement) with reduced thermal conductivity, precluding effective heat dissipation. In the past, an impressive research effort has been devoted to photon and electron management in Si, both bulk and nanocrystalline, but also phonon management should not be neglected. Indeed, following photon absorption, free carriers with excess energy typically appear; their subsequent cooling to band edges generates phonons. In case of bulk materials, (acoustic) phonons effectively remove the energy from the place where the photon absorption took place. However, when this process of heat transport by phonons becomes inefficient, the presence of phonons can significantly alter the physical and optical properties of the system. Especially at interfaces and/or in nanostructures, phonon localization and trapping cannot be ignored, since these can alter the vibrational lifetimes considerably^15^. It has been shown that the thermal conductivity of bulk Si can efficiently be reduced by using nanostructures of 10–100 nm in size^16^,^17^. Also, scattering processes at interfaces play a significant role in nanostructures^16^,^17^. In case of Si nanocrystals, it has been shown that the thermal conductivity changes upon embedding, being significantly different for free-standing Si particles and those dispersed in a host matrix: experiments clearly demonstrated that the former could easier be heated up by continuous wave laser illumination than the latter, due to their relatively poor thermal conductivity^13^,^18^,^19^,^20^.

Here, we report on superlinear photoluminescence (PL) emission in the visible and infrared range, obtained with crystalline Si NP’s deposited with high layer porosity. In fact, enormous yield enhancement is achieved in this spectral range, with an amplification gain of up to several orders of magnitude, which is size- and shape-dependent and correlated to the layer porosity.

## Experimentals

### Materials

For the purpose of this study, an inductively coupled plasma chemical vapor deposition reactor is used to synthesize large Si NP’s with dimensions in the tens to a few hundreds of nanometers range, so too large for the quantum confinement effect to play a role, and with a narrow size distribution (FWHM <5%). Depending on the plasma parameters (e.g. pressure, deposition time, radiofrequency applied, gas ratios) Si NP’s of different shapes as well as different sizes can be produced (see Methods for more details). This procedure does not require high temperatures during the formation (the substrate is maintained at a temperature of only 50 °C) nor subsequent annealing, offering easy and cheap deposition of high crystalline quality large Si NP’s on any substrate. The layer thickness can be varied from a sub-monolayer of Si NP’s up to a few micrometers. In this study, four types of materials have been investigated, featuring regular octahedral Si NP’s and irregular ones.

### Characterization

**Figure 1:**
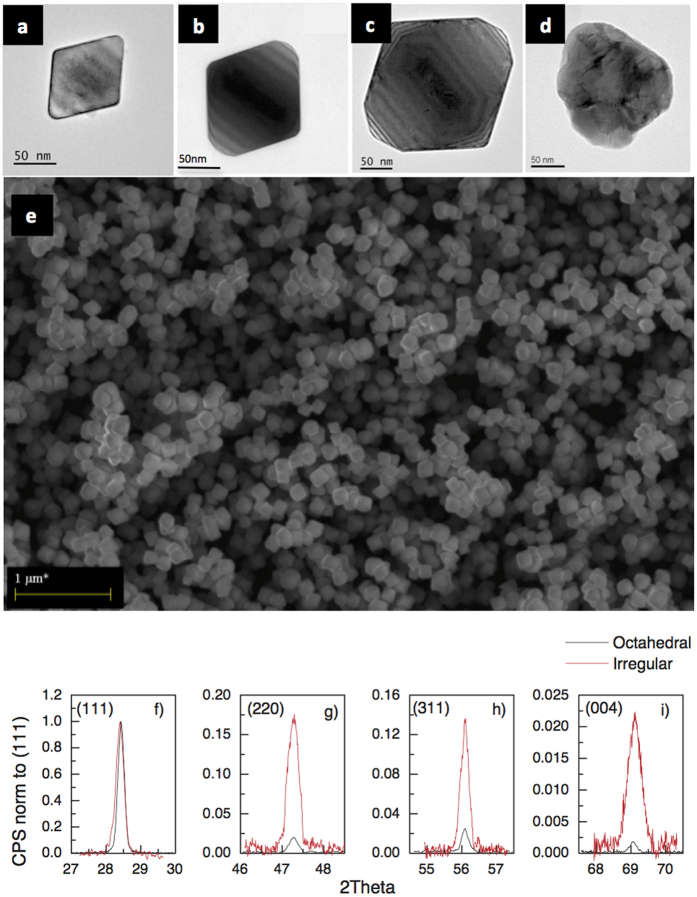
Sample characterization. (**a**–**d**) Transmission electron microscopy (TEM) images of the octahedral (**a**–**c**) and irregular Si NP’s (**d**). The size of the octahedral Si NP’s (80 nm, 110 nm, 140 nm) is determined as the length of 220 plane (edge of the triangular facet). For the irregular Si NP the size is the particle average diameter. Note that the octahedral Si NP’s in (**b**) and irregular Si NP’s in (**d**) have very similar size. (**e**) Scanning electron microscope (SEM) plan view of a 2 μm thick layer formed by octahedral Si NP’s. (**f**–**i**) X-ray diffraction (XRD) Bragg peaks related to the (111), (220), (311), (004) for octahedral (black) and irregular (red) Si NP’s. All the octahedral NP’s show the same peak width and intensity after normalization. In the figure, the intensity of the peaks was normalized to the (111) contribution, in order to highlight the nonrandom distribution of the octahedral Si NP’s. Complete XRD spectra with markers are included as Supplemental Information.

The differences between the four types of investigated materials are directly confirmed by transmission electron microscopy (TEM) images of the ~80 nm, ~110 nm, ~140 nm octahedral Si NP’s – Fig. 1(a–c) – and ~110 nm irregular Si NP’s – Fig. 1(d). From the scanning electron microscopy (SEM) image (Fig. 1(e)), the layer porosity can be clearly attested by the presence of well separated columnar structures of NP’s and the consequent formation of pores beneath the surface. In most cases these pores are not observable by SEM, being fully covered, and have been detected and quantified by Coherent X-ray Diffractive Imaging (CXDI), as discussed in the following. From X-ray Diffraction (XRD) analyses – Fig. 1(f–i) – taken on irregular and on a generic octahedral NP, it is evident that the actual shape and size of the Si NP’s do not have a strong influence on their crystal quality (see Supplementary Fig. SI1 for a full XRD spectrum with markers). However, it was further observed that for all the three octahedral Si NP sizes, the relative intensity of the peaks, normalized to the (111) value, diverges significantly from the values expected in a randomly oriented polycrystalline Si layer^14^. This finding derives from geometrical consideration, since the octahedral Si NP’s more likely expose upwards the larger (111) facets than all the other smaller planes. In contrast, the same analysis for irregular Si NP’s yields values similar to those expected for a polycrystalline Si layer, with randomly oriented crystallites, due to the lack of preferential landing planes. As shown in the following, the layer porosity, modulated by either the modification of the size or shape of the Si NP’s, strongly determines the phonon trapping, with a consequent increase of local temperature and a strong PL emission.

## Discussion of Experimental Results

### Photoluminescence in the visible

**Figure 2:**
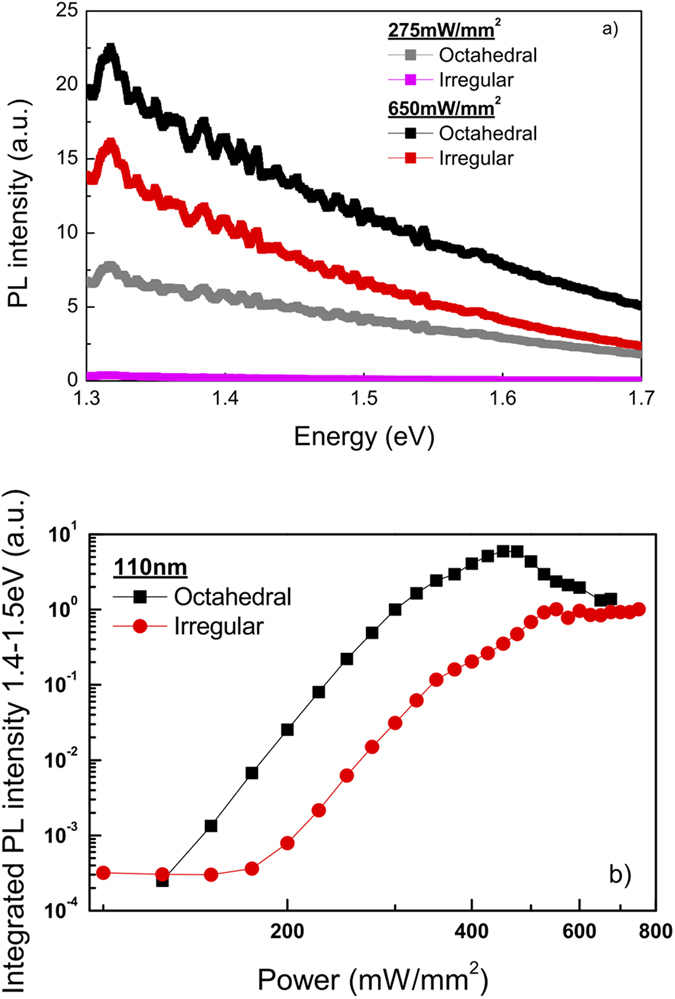
Photoluminescence in the visible detection range. (**a**) Typical PL spectra obtained under 2.33 eV continuous wave excitation for ~110 nm octahedral (black) and irregular (red) Si NP’s in the range 1.3–1.7 eV for two different laser excitation powers. (**b**) Power dependence of intensity of the two samples under 2.33 eV continuous wave excitation depicted in double logarithmic representation. The integrated PL intensity is determined by integrating the PL spectra – Fig. 2(a) – between 1.4 and 1.5 eV. The power dependences of both samples, depicted in double logarithmic representation, show a superlinear behavior with a slope up to ~10 and a four order of magnitude enhancement in the investigated power range.

A typical PL spectrum observed for a layer of ~110 nm octahedral Si NP’s under 2.33 eV continuous wave excitation is shown in Fig. 2(a) – black curve – with the corresponding power dependence of its intensity in Fig. 2(b), depicted in double logarithmic representation. From the latter, we conclude that the PL intensity increases superlinearly with the laser power (slopes of up to ~10), with an intensity enhancement exceeding four orders of magnitude for the investigated laser power range. We note that the PL intensity decreases for the highest powers; this is related to the permanent damage inflicted on the sample by the intense laser beam.

In order to investigate if the PL spectrum is dependent on the particle shape, results of similar measurements for a layer of ~110 nm irregular Si NP’s have been obtained and are shown for comparison in Fig. 2, in red. The PL spectrum as well as its power dependence are similar as for octahedral Si NP’s, but with a relatively lower intensity for the same power setting. Besides the shape-invariance of PL, the emission was also found to be independent of a particular substrate (not shown). This is particularly important for the possible application potential, showing that Si NP’s with nearly identical emission properties can be deposited on different substrates.

### Photoluminescence in the near infrared

**Figure 3:**
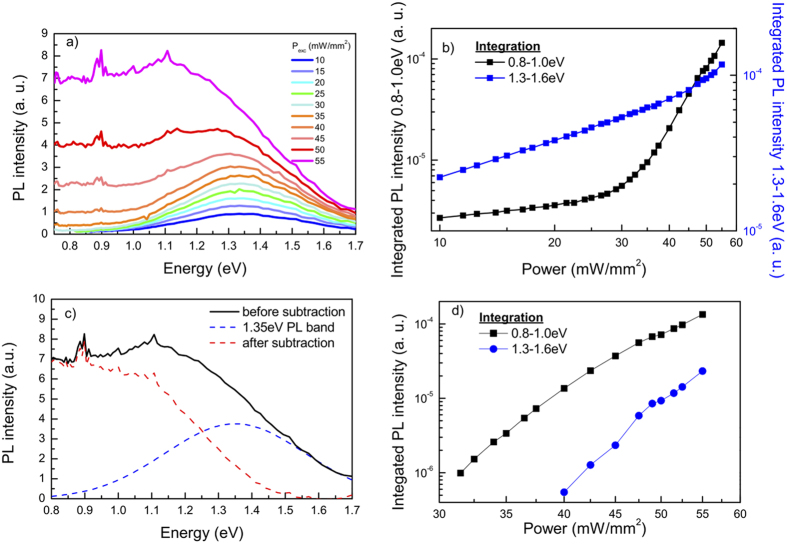
Photoluminescence in the (near) infrared detection range. (**a**) Typical PL spectra of ~110 nm octahedral Si NP’s under 2.33 eV continuous wave excitation, for several values of the excitation power (P_exc_) using a more sensitive detector with a broader detection range than in Fig. 2. (**b**) Power dependence of the integrated PR intensity between 0.8 to 1.0 eV (black, left y-axis) and 1.3 to 1.6 eV (blue, right y-axis) under 2.33 eV continuous wave excitation depicted in double logarithmic representation. (**c**) Example of the resulting emission (red) after subtraction of the PL band centered around ~1.35 eV (blue) from the total measured emission (black). (**d**) Power dependence of the 0.8 to 1.0 eV (black) and 1.3 to 1.6 eV (blue) ranges after subtraction of the PL band centered around ~1.35 eV following the procedure as depicted in panel (**c**).

The PL measurements were extended to the (near) infrared detection range, using much lower irradiance and a more sensitive detector than for the experiments in the visible detection range (which are shown in Fig. 2). Figure 3(a) shows typical PL spectra of one of the samples (~110 nm octahedral Si NP’s) under different irradiances. One should note that the excitation powers cannot be directly compared to those of Fig. 2, because of differences in the experimental procedures (see also Methods). For the lowest excitation levels, a Gaussian-shaped emission band can be distinguished, centered around ~1.35 eV. With increasing excitation power, the intensity of this PL band grows linearly (slope limited to values <1 on the double logarithmic scale, see also Fig. 3(b)), while its peak position remains constant. Under higher excitation power, another broad emission band appears at the low energy side, in the 0.8–1.0 eV range. In contrast to the 1.35 eV PL band, the intensity of this new emission grows superlinearly with the laser power. This superlinearly increasing emission appears first in the lower energy range, but due to its rapid growth affects also the high energy part of the spectrum, at higher pump powers: this is illustrated in Fig. 3(b), which shows the laser power development of PL intensity integrated for two different energy ranges. In this case, the change of the slope can be seen, indicating the regions where each PL band dominates the integrated emission.

We conclude that the PL of the investigated samples originates from at least two channels of radiative recombination. The shape of the PL band centered around ~1.35 eV is independent of the laser power (normalized spectra for that band are identical, not shown) while its intensity increases linearly. This PL band, which dominates emission at low excitation levels, could originate from a small amount of amorphous Si, as identified at low temperature by Kanemitsu^21^, since during the sample fabrication process amorphous Si could have been deposited. To determine the origin and the characteristics of the other emission channel(s) more thoroughly, we separated the band centered around 1.35 eV and “subtracted” its contribution from the total PL spectra. In that procedure we have assumed the linear power dependence and position and shape invariance of the 1.35 eV PL band for the whole investigated power range (see Fig. 3(c) for an example). For every single PL spectrum, we followed the procedure depicted in Fig. 3(c). Integrating all the remaining PL for the investigated powers again between 0.8 to 1.0 eV and 1.3 to 1.6 eV and plotting the results as a function of pump power – Fig. 3(d) - reveals now slopes of approximately 10 for both ranges: similar as in Fig. 3, the behavior is superlinear.

### Temperature of the emitting sample

**Figure 4:**
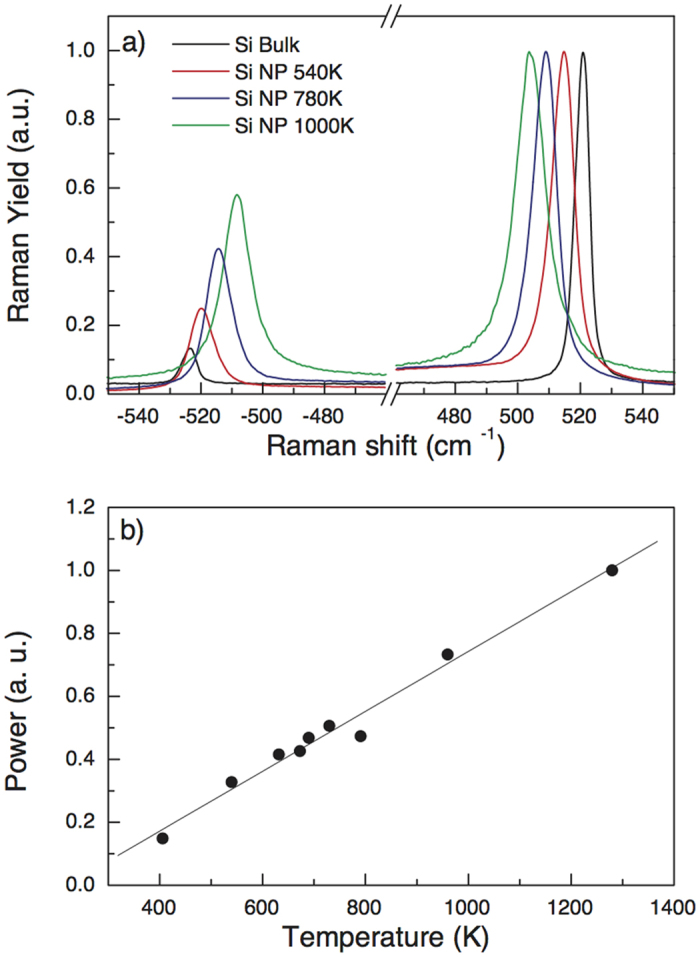
Raman spectroscopy. (**a**) Micro-Raman spectra obtained in bulk Si and a layer of Si NP’s, using 1.96 eV laser continuous wave excitation. All data are normalized to Stokes peaks. Both peaks, Stokes and anti-Stokes, are shown, permitting the determination of local temperature in each sample. A shift of about −15 cm^−1^ is detected at about T = 1000 K. (**b**) Plot of the temperature dependence versus laser power illuminating the sample.

Raman spectroscopy can provide a wealth of information on low-dimensional systems, e.g., on the chemical composition, structure and thermal conductivity^22^,^23^, and in the present case also allows to establish the temperature at which this strong PL emission occurs. In Fig. 4(a), Raman spectra of the Si NP’s, featuring both Stokes and anti-Stokes peaks, are compared at different temperatures to that of bulk Si. We observe a large shift of Stokes and anti-Stokes peaks, (respectively about 1.895 eV and 2.023 eV, at RT) and a broadening of their line width with respect to bulk Si. From the intensity ratio of the two peaks, the local temperature of the Si NP’s illuminated by the laser beam can be determined as:


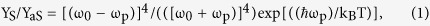


where Y_S_ and Y_aS_ indicate the yield of the Stokes and anti-Stokes peaks, respectively, ω_0_ is the laser frequency, ω_p_ the phonon frequency, k_B_ the Boltzmann constant, ℏ the reduced Planck constant and T the local temperature (see ref. 13 for further details on the determination of the local temperature). For the layer of ~110 nm octahedral Si NP’s it has been established that the damage takes place when the sample temperature is higher than ~1000 K. Naturally, reducing the laser power, also the temperature decreases, as shown in Fig. 4(b).

Raman measurements on the same sample (making use of Eq. 1) and previous work^13^ reveal that the local temperature of the Si NP’s can rise up to ~1350 K under continuous wave laser excitation at 1.96 eV. This agrees with our previous investigations on similar materials^13^. Also other works showed that free-standing Si NP’s of different sizes can be heated due to laser illumination^18^,^19^,^20^. Therefore it is reasonable to expect that in our PL experiment, with the continuous wave excitation set to 2.33 eV, the Si NP’s will heat up significantly. Indeed, the results of the PL measurements in the visible range certify that the heat generation is so high that the material alters, leading to a decrease of PL intensity. In addition, no excitonic emission from the large Si NP’s was observed under low power excitation, so at low temperature (close to RT). Therefore, it is very likely that heating plays a major role in generation of the PL observed under high power excitation. Moreover, we found that a very high irradiance is necessary in order to observe emission from these samples under nanosecond pulsed laser excitation, and that the material in this case deteriorates very quickly (within less than a second, not shown). This is readily explained by the very high peak powers in that excitation mode, leading to intense heat generation.

### Origin of the strong photoluminescence

From the just discussed observations, we conclude that the PL is being emitted while the investigated sample attains high temperature. Therefore one possible origin of the strong PL could be emission due to indirect electronic transitions fostered by the high phonon concentration, due to their confinement in Si NP’s, as suggested before in ref. 13. To this end, we recall that the (indirect) bandgap of Si reduces significantly at higher temperature^24^, with the effect being further magnified by including electron-phonon interaction^25^. The detected emission bands in Fig. 3(a) strongly support the contribution of indirect electronic transitions.

Besides, blackbody radiation of the Si NP’s could also play a role^18^,^19^. According to Planck’s law, the blackbody radiation of an object as function of the emission wavelength and its temperature can be described as:


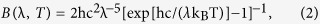


where B denotes the wavelength- (λ) and temperature- (T) dependent blackbody emission, h Planck’s constant, c the speed of light in the medium, and k_B_ Boltzmann’s constant. The maximum intensity of the blackbody radiation spectrum moves to the higher energies with increasing temperature and its intensity at a certain energy shows a superlinear temperature dependence (see Fig. 2 in Supplementary Information). For the highest temperature reached in our measurements, ~1350 K, the peak of the blackbody radiation spectrum should move to ~0.58 eV, still outside of our detection range. For a certain temperature of the body, the slope on the double-logarithmic scale increases with detection energy, similar as observed in this study – Fig. 3(d).

Since both discussed radiative recombination processes are strongly dependent on the temperature and can occur simultaneously and independently of each other, we propose that they both contribute to the experimentally observed strong PL enhancement. For both processes, the capability of the Si NP’s to exchange and dissipate energy should be essential, thus influencing the emission. This, in turn, should depend on the effective density of Si NP’s in the layer, realized by grain agglomeration during deposition.

### Effect of porosity

**Figure 5:**
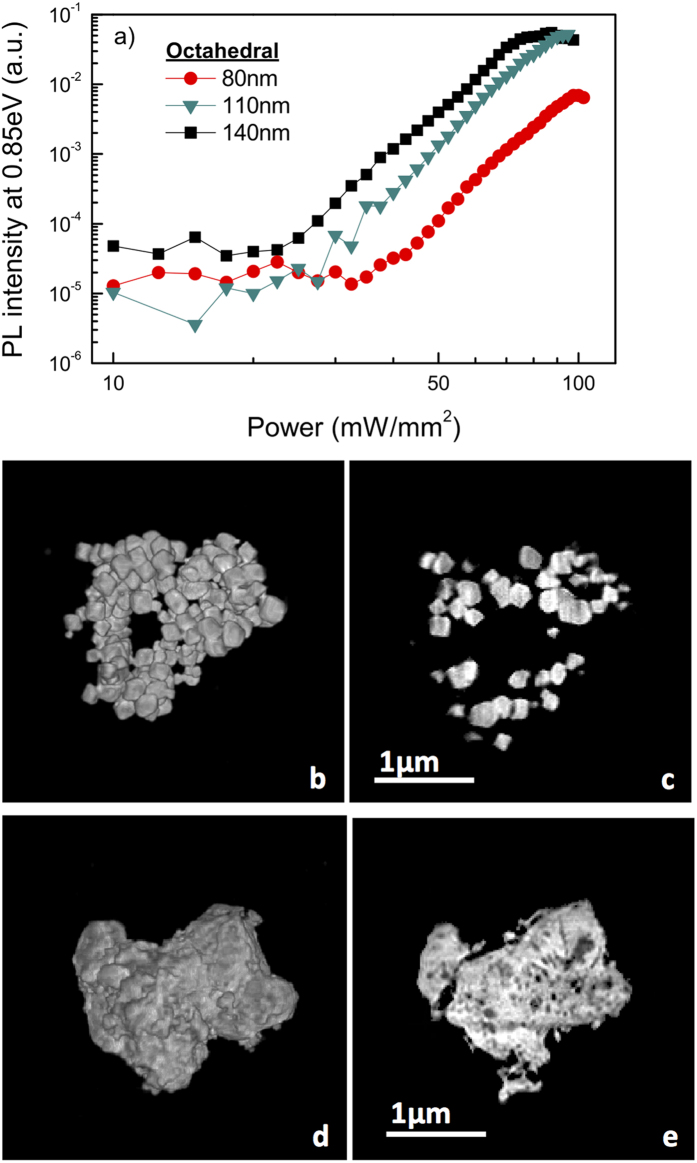
Porosity. (**a**) Superlinear PL yield, as obtained at 0.85 eV, for the octahedral Si NP’s of three different sizes (~80, 110 and 140 nm) under 2.33 eV continuous wave excitation. (**b**) Volume representation and (**c**) central slice of a sample formed by agglomerated octahedral Si NP’s. (**d**) Volume representation and (**e**) central slice of a sample made from the agglomeration of irregular Si NP’s. (**e**) The central slice of the irregular Si NP’s showing the degree of compactness and the noticeable smaller porosity.

In order to confirm the essential role of the sample temperature in the observed PL enhancement, we investigate the effect of energy exchange within the ensemble of the Si NP’s and dissipation to the environment. For that we have first measured PL intensity of three different sizes of octahedral Si NP’s, namely ~80 nm, ~110 nm, and ~140 nm. All three samples have the same amount of Si deposited and show a superlinear power dependence of PL intensity as reported in Fig. 5(a), where the PL intensity is directly proportional to the NP size for the same power setting. We conclude that the PL intensity is dependent on the shape (see Fig. 2) as well as the size (Fig. 5(a)) of the NP’s, indicating that the porosity of the material is an important parameter. Consequently, we have investigated the agglomeration of the Si NP’s during the deposition. We have studied the spatial configuration of the different types of Si NP’s employing Coherent X-ray Diffractive Imaging CXDI technique. This three-dimensional (3D) high resolution novel lens-lens imaging technique is based on the numerical inversion of scattering patterns measured under coherent illumination in the far-field regime and it exploits the degree of coherence of modern synchrotron sources^26^. Images of the octahedral as well as the irregular Si NP’s were reconstructed using this novel technique, showing individual Si NP’s and their internal porosity with unprecedented detail – Fig. 5(b–d). As can clearly be seen, the deposition of the Si NP’s produces layers whose porosity depends on the Si NP’s size and shape. The CXDI measurements allow retrieving the 3D electron density distribution of the sample expressed in grey level values. The “porosity” parameter p (0 < p < 1) of the specific deposition can be extracted simply from the 3D reconstructions as the average deposition density (or average grey level value), scaled to the bulk Si density, according to the formula p = 1 − ρ_sample_/ρ_bulk_, where ρ_sample_ and ρ_bulk_ are the sample and the bulk densities, respectively. The bulk intensity is estimated by histogramming the grey levels in each single slice. In the case of the octahedral, where sections of individual Si NP’s are clearly distinguishable – Fig. 5(b), the determination of the average grey level representative of the bulk is trivial. For the sample of irregular Si NP’s, this operation is more difficult, since images of individual Si NP’s are not resolved – Fig. 5(d). However, the grey level histograms show a quite narrow Gaussian distribution in the homogeneous areas with a nearly constant average value over all slices. We assume this value to be representative of the bulk porosity of the sample with irregular NP’s. The average grey level of the whole sample volume is evaluated by normalizing the average value of the grey level histogram of the sum of the slices to the number of slices representative of the sample thickness. Not surprisingly, the two samples show quite a different porosity – Fig. 5(b–d). For octahedral Si NP’s, the average value of the porosity is p = 0.67 with a minimum value around 0.55 in the most compact parts; for irregular Si NP’s we find p = 0.41, with the minimum value around p = 0.16 in the most dense parts (see Table 1). Since the sample with octahedral Si NP’s has a higher porosity than the one with irregular Si NP’s, the inter-NP heat conductivity is lower for the former than for the latter. Considering the direct relation between heating and PL intensity, this is consistent with the systematically lower PL intensity observed for the irregular Si NP’s (Fig. 2). We note that scaling the PL intensity to the illuminated volume of the emitter will increase the PL intensity difference even more, since the sample with irregular NP’s has a lower porosity, while the spot size and layer thickness were identical.

## Conclusions

Intense emission in the visible as well as the (near)-infrared range is obtained from large Si NP’s deposited as a porous layer, with individual NP’s being in poor contact with each other. The Si NP’s are too large for quantum confinement effects to play a role and no excitonic emission is observed at room temperature. Nevertheless, strong PL with superlinear power dependence of its intensity is found, with slopes up to ~10 in the double logarithmic representation. We propose that the significant increase of the temperature of Si NP’s, appearing due to the reduced heat conductivity and the “phonon confinement” within the Si NP’s, is responsible for this emission. Measurements on materials with different sizes as well as different shapes NP’s show that the PL intensity can be directly linked to the porosity of the layer. In this way, PL characteristics can be tuned by the porosity of the grain layers, which control heat exchange between individual Si NP’s, and the overall heat conductivity of the whole layer, opening interesting application fields of this unique form of Si-based material.

## Methods

### Sample preparation

Samples were prepared in vacuum (5 × 10^−7^ Torr) in an inductively coupled plasma chemical vapor deposition reactor exploiting the capability of this non-thermal plasma to the enhance Si crystalline particle formation for the SiH_4_/Ar gas mixture, rather than the SiH_4_ decomposition into an amorphous Si deposited layer. This reactor has a vertical geometry, the plasma is highly ionized (~5 × 10^11^ cm^−3^) and the substrate was maintained at low temperature (50 °C).

Deposition is performed on 6 inches diameter substrates using the same precursor gas ratio for 15s (Fig. 1(a)), 45s (Fig. 1(b)) and 90s (Fig. 1(c)). The variation of the shape from octahedral to irregular Si NP’s (Fig. 1(d)) is obtained by simply adding 15% H_2_ during the plasma ignition. Very likely, a large amount of H_2_ present in the deposition chamber alters the Si NP’s formation reactions, similarly to what it is described in refs 27 and 28, inhibiting the formation of a single crystal (in our case the octahedral Si NP’s). Finally, performing the process for a specific time and repeating the step as many times as needed up to the desired density and layer thickness controls the density of NP’s up to the complete coverage of the substrate as it is the case of the Fig. 1(e).

### Sample characterization

The samples were analyzed by using a ZEISS SUPRA35 scanning electron microscopy (SEM), equipped with a field emission electron gun and by transmission electron microscopy (TEM) using a JEOL JEM 2010 microscope with a LaB6 thermionic source, operating at an acceleration voltage of 200 kV and equipped with a Gatan multi-scan digital camera. We used a Bruker-AXS D8Discover diffractometer equipped with a CuKα source and soller slits as thin film attachment; the diffraction patterns were acquired in the symmetric geometry, with the source and the detector symmetrically moving with respect to the sample surface.

The morphology of the clusters was studied by CXDI at the beamline ID10 of the European Synchrotron Radiation Facility. CXDI is a lens-less imaging technique where the electron density distribution of an isolated object in real space is retrieved by an iterative algorithm that phases an oversampled speckle pattern recorded in reciprocal space in the far field. The resolution in real space is determined by the highest Q-vector where the scattered signal can be recorded with a sufficient signal-to-noise ratio 26. Micrometric sized Si NP’s were extracted from the samples and deposited on Si_3_N_4_ membranes. The 2D diffraction data were taken by a MAXIPIX pixel detector (516 × 516, 55 micrometer pixel size) by using a 8.1 keV, 10 micrometer coherent X-ray beam and rotating the Si NP’s over an angular range 150° and 160° with 0.5° angular increment. The first data treatment (background subtraction, dead-time and flat-field correction) and the assembling of the 3D Fourier space were performed as described in ref. 29. The image reconstructions were obtained through the iterative hybrid input-output phase retrieval algorithm by the in-house software. Several solutions from different random starts were averaged to obtain the final result. The high quality of the 3D reconstructions with 17.9 nm voxel size reveals individual Si NP’s and their internal porosity with unprecedented detail. The samples were also investigated by micro Raman spectroscopy for detecting the transversal optical (TO) vibrational peak situated for bulk Si at 521 cm−1, at room temperature (~300 K) in air. Raman spectra are collected in backscattering geometry with a HORIBA Jobin-Yvon system, equipped with Olympus BX41 microscope. He-Ne laser radiation at a wavelength of 1.96 eV is focused by a 100× objective. The laser power on the sample is 6 mW, and a 550 mm focal length spectrometer with 1800 lines/mm grating is used for collecting Raman spectra.

### Photoluminescence measurements

Photoluminescence spectra were measured in the visible as well as the (near)-infrared detection range under 2.33 eV continuous wave laser excitation (Millenia IIs laser system). Given that NP’s are covered with native oxide and the heating maximum temperature under laser irradiation is around 900 °C (above that we observed layer damage), we estimated a minimal oxide increase of only few Angstroms, not influencing the results. In the visible regime (Fig. 2), a thermoelectric-cooled charge-carrier device (CCD, Hamamatsu S101141-1108S) coupled to a spectrograph (Solar M266) is used, while a liquid-nitrogen cooled Ge photodiode (Edinburgh Instruments) with a chopper and a DSP lock-in amplifier (Signal Recovery SR7265) coupled to a Jobin-Yvon THR-1000 spectrometer is used in the (near) infrared detection range (Figs 3 and 5(a)). The excitation flux for the measurement shown in Fig. 2 is significantly higher than that of Figs 3 and 5(a). The laser power on the sample is ~0.2–1.5 W with a spot size of ~2 mm^2^, ~0.1–0.55 W with a spot size of ~10 mm^2^ and ~0.1–1 W with a spot size of ~10 mm^2^ for Figs 2,3 and 5(a), respectively. Since both the PL intensity and the sample deterioration are actually governed by thermal effects and the actual temperature reached by the sample, we cannot easily scale the excitation fluxes between Figs 3 and 5(a) (both measured with Ge detector) and Fig. 2 (measured with CCD). In view of differences in the experimental procedures, the heat management within the sample will be dissimilar leading to a different sample temperature for nominally the same flux values. All spectra are corrected for the sensitivity of the experimented setup and measured at room temperature in air.

## Additional Information

**How to cite this article**: Jong, E.M.L.D. *et al*. Strong infrared photoluminescence in highly porous layers of large faceted Si crystalline nanoparticles. *Sci. Rep.* **6**, 25664; doi: 10.1038/srep25664 (2016).

## Supplementary Material

Supplementary Information

## Figures and Tables

**Table 1:** Porosity.

	Average porosity	Minimum porosity
Octahedral	0.67	0.55
Irregular	0.41	0.16

Porosity of the ~110 nm octahedral and irregular Si NP’s.
