# Supplementary material for: Strong infrared photoluminescence in highly porous layers of large faceted Si crystalline nanoparticles
Source: Sci Rep. 2016 May 24;6:25664. doi: 10.1038/srep25664 (PMC4877587; doi:10.1038/srep25664)
Supplement: Supplementary Information [file srep25664-s1.pdf]

Strong infrared photoluminescence in highly porous layers of large faceted Si crystalline nanoparticles

E.M.L.D de Jong[1], G. Mannino[2,*], A. Alberti[2], R. Ruggeri[2], M. Italia[2], F. Zontone[3], Y. Chushkin [3], A.R. Pennisi[4], T. Gregorkiewicz[1], and G. Faraci[4]

[1] Van der Waals – Zeeman Institute, University of Amsterdam, Science Park 904, 1098 XH Amsterdam, The Netherlands

[2] IMM – Consiglio Nazionale delle Ricerche (CNR-IMM), VIII Strada n°5 Zona Industriale, 95121 Catania, Italy

[3] ESRF, the European Synchrotron, CS40220, 38043 Grenoble Cedex 9, France

[4] Dipartimento di Fisica e Astronomia, Università di Catania, Via Santa Sofia 64, 95123 Catania, Italy

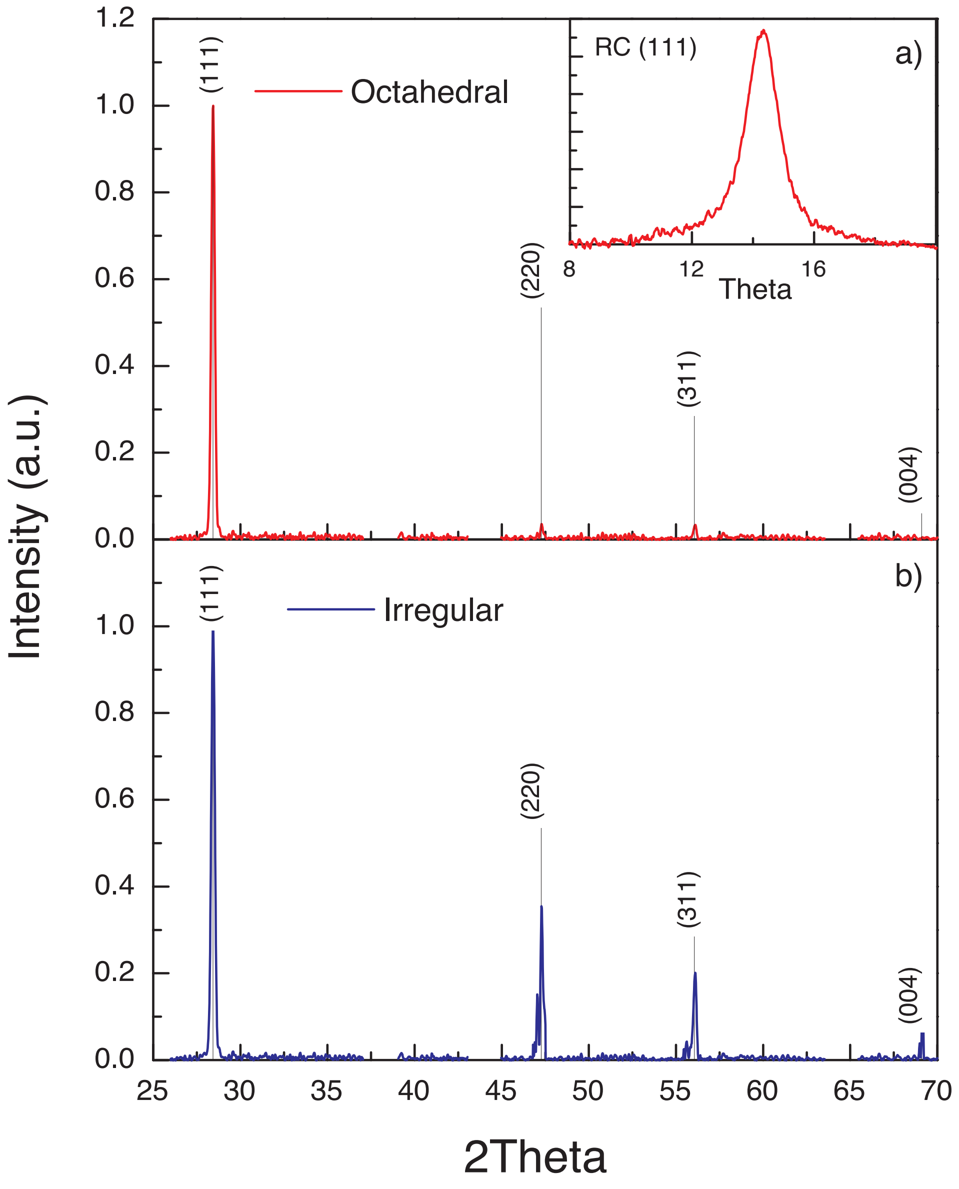

Figure SI1

Full XRD diffraction pattern with markers.

(a) X-ray diffraction pattern taken in symmetric configuration on a layer formed by octahedral nanoparticles like those in Fig. 1(a-c). With this method, the crystallographic planes lying parallel to the sample surface have selectively contributed to the collected signal. Both the diffraction patterns and the reference bars of the random powder are normalized to one. The comparison evidences a high degree of texturing of the whole system along the [111] direction. This texturing is further reinforced by the intense rocking curve (RC, see inset) relative to the (111) planes (FWHM 1.6°). All those findings are in agreement with the description given in the text.

(b) X-ray diffraction pattern taken in symmetric configuration on a layer formed by irregular nanoparticles like that in Fig. 1(d). This sample has a minor texturing degree.

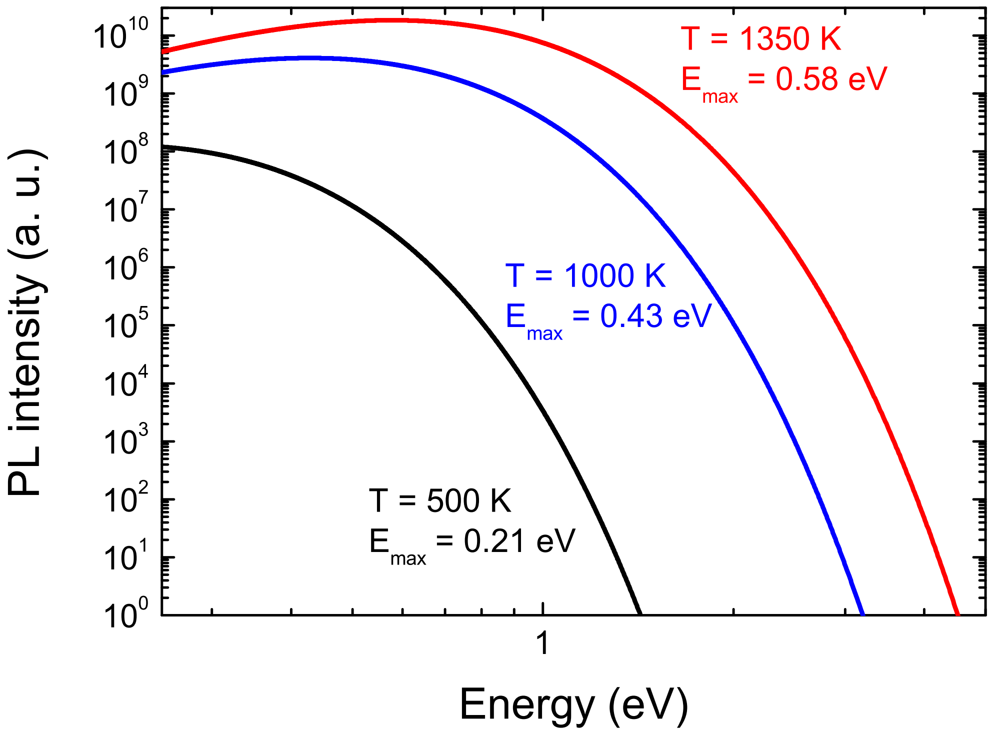

Figure SI2

Blackbody radiation.

Thermal emission, at the indicated temperature, as a function of energy following from Eq. 2. For each temperature the maximum of emission, following Wien's law, is also reported. No band is present in the range of interest.
